# Hiding in plain sight: children with visual perceptual difficulties in schools

**DOI:** 10.3389/fnhum.2024.1496730

**Published:** 2024-11-27

**Authors:** Nicola McDowell, Helen St Clair Tracy, Andrew Blaikie, John Ravenscroft, Gordon N. Dutton

**Affiliations:** ^1^Institute of Education, Massey University, Auckland, New Zealand; ^2^Infection and Global Health Division, School of Medicine, University of St Andrews, St Andrews, United Kingdom; ^3^The Scottish Sensory Centre, Moray House School of Education and Sport, University of Edinburgh, Edinburgh, United Kingdom; ^4^Department of Vision Sciences, Glasgow Caledonian University, Glasgow, United Kingdom

**Keywords:** cerebral visual impairment, visual perceptual difficulties, children and adolescents, screening and assessment, education support

## Abstract

Cerebral visual impairment (CVI) is increasingly being recognized as a significant cause of visual difficulties in children, particularly those with typical visual acuity, who nonetheless struggle in educational settings. This narrative review aims to elucidate the nature and impact of visual perceptual difficulties (VPD) associated with CVI in school-aged children, who often remain undiagnosed due to the current erroneous focus on visual acuity as a required diagnostic criterion for CVI. The review synthesizes findings from recent studies, highlighting that up to 3.4% of children in mainstream schools and a higher percentage in special educational settings may experience VPD, which significantly impacts upon their learning and development. The manifestations of VPDs, such as difficulties in motion perception, recognition, and visuospatial processing, are often subtle and can thus be overlooked, leading to misconceptions about the origins of the affected child’s abilities and behaviors. The review also discusses the challenges in current diagnostic processes, emphasizing the need for comprehensive history taking and assessments that go beyond standard visual acuity tests. It proposes a multi-faceted approach to identification and support, incorporating both clinical assessments and teacher/parental observations, to better address the needs of affected children. Furthermore, this paper advocates for the inclusion of VPDs in the International Classification of Diseases (ICD 11) to ensure children with these visual issues receive appropriate educational support. By integrating lived experiences of individuals with CVI and the latest research findings, this review underscores the urgent need for awareness and tailored educational strategies designed to support children with VPDs. The findings suggest that without such recognition and intervention, many children with VPDs will continue to “hide in plain sight,” facing unnecessary challenges in their educational and social development. The review concludes with recommendations for policy changes and future research directions to improve the identification, classification, and support of children with VPDs within the educational system.

## 1 Introduction

Cerebral visual impairment (CVI) is a term used to describe a wide range of visual difficulties due to damage or injury to the visual areas of the brain (Lueck and Dutton, 2015). Unlike visual problems caused by issues with the eyes or the optic nerves (the pathways that carry visual information from the eyes to the brain), CVI arises from dysfunctions in the brain’s ability to process what is seen. People with CVI may experience various visual challenges, such as reduced clarity of vision, difficulty seeing in certain parts of their visual field, problems with eye movement control, and/or challenges in recognizing and interpreting visual information (Williams et al., 2021). One common outcome of CVI is visual perceptual difficulties (VPDs). VPDs refer to the brain’s struggle to make sense of visual information, which can lead to issues like difficulty recognizing objects or people, understanding spatial relationships, issues with visual attention and processing moving and/or complex visual scenes (Chandna et al., 2021). Despite over a century of awareness about VPDs, with early reports of visual issues observed in soldiers returning from World War I (Riddoch, 1917; Holmes, 1918a; Holmes, 1918b), there is still widespread confusion and misunderstanding surrounding these difficulties. While there have been significant advancements in the past decade which is helping to reduce some of this confusion, much of this information is still being disseminated to professionals working in both the education and health sector. This means that for many, CVI and VPDs are still relatively unknown conditions, which often leads to the mismanagement of children and adolescents with these visual issues. This lack of understanding can be detrimental, especially for those who may struggle in school due to their undiagnosed visual issues related to CVI, which may also lead to inappropriate behavioral diagnostic labelling.

Studies have shown that VPDs are not uncommon in children. For instance, research indicates that about 3.4% of children in mainstream primary schools have visual difficulties related to CVI, even though most have typical visual acuity (Williams et al., 2021). The prevalence is even higher in special schools, where between 23.5 and 58% of children may experience CVI-related visual challenges, often without having a formal diagnosis (Black et al., 2019; Williams et al., 2021). Lueck and Dutton (2015) categorized children affected by CVI into three broad groups: those who attend mainstream schools with typical peers, those who face learning difficulties, and a third group who are the most profoundly affected. Even in this third group, where visual impairments are more easily recognized due to reduced visual acuity, unrecognized VPDs are often a significant part of their challenges (Little and Dutton, 2014). Understanding and addressing VPDs is crucial for providing appropriate support to children with CVI, ensuring they can better engage with their learning environments.

This review aims to explain how to better understand and assist children who struggle with VPDs. By firstly examining both research and personal accounts, we will explore how these difficulties show up in daily life and how they can affect a child’s learning and development. We will also discuss the ongoing debate around how CVI is defined, particularly in relation to children in both mainstream and special schools. This is important because many children with these visual issues have typical or near typical visual acuity, yet they still face significant challenges in accessing and interpreting learning material (Tsirka et al., 2020). Given the high number of children affected, this is a critical issue. We will look at how well current systems for assessment, diagnosis, and support are meeting the needs of children with CVI and whether there are better ways to understand and address VPDs. Our goal is to highlight that the current approach may not be sufficient and that more needs to be done to ensure children with CVI are not overlooked. In the discussion section we highlight how drawing on the knowledge from researchers, clinicians, educators and those with the lived experience, and working together, will allow the field to find practical and achievable steps to better identify, and support affected children.

## 2 Unpacking visual perceptual difficulties

The earliest documented reports of brain related visual difficulties describe a range of visual issues. These include those of the primary visual functions, low visual acuity and visual field deficits (Holmes, 1918a). Reported VPDs include an inability to see more than one of two objects at the same time (simultanagnosia), impaired visually guided movement (optic ataxia), inability to volitionally direct visual gaze (apraxia of gaze), further disturbances of ocular movement, inability to recognize objects, letters, faces, difficulty with reading and writing, disturbances of visual localization and orientation in space and reduced ability to see movement (Bálint, 1909; Holmes, 1918a; Holmes, 1918b; Guzzetta et al., 2009), while recovery of the ability to see only movement after visual brain injury (Riddoch, 1917) has also been described (Arcaro et al., 2019). The work by Goodale and Milner (2013) has been foundational in understanding the visual processing pathways, specifically the theories of the dorsal and ventral streams, often referred to as the ‘where’ and ‘what’ pathways. The dorsal stream, responsible for vision for action, runs from the occipital lobe to the posterior parietal cortex at the top of the cerebral hemispheres. In contrast, the ventral stream, which is involved in vision for perception, extends from the occipital lobe to the lower and lateral regions of the cerebral hemispheres in the inferior temporal lobes. More recently the anatomical correlates with the dorsal and ventral stream pathways have been identified, respectively, as being the superior and inferior longitudinal fasciculi (Bennett et al., 2020).

VPDs are often linked to atypical processing in the posterior parietal lobes and are sometimes referred to as dorsal stream dysfunction. While this term is often applied to children experiencing additional reduced acuity and/or visual processing challenges (Dutton, 2009), it’s application may be less accurate for those children who present with typical visual acuities, yet still face difficulties in visual processing, such as recognizing faces or objects. The anatomical correlate of the dorsal stream, the superior longitudinal fasciculus, connects the occipital, posterior parietal and frontal lobes. If the occipital lobes are functioning typically, it is therefore, plausible that any dysfunction may arise elsewhere in the pathway, particularly in the posterior parietal regions. This raises the possibility that what is traditionally labelled as dorsal stream dysfunction may, in some cases, involve impairments unrelated to the occipital lobe functions, suggesting a more complex interplay of brain networks. This further supports the view of Merabet and Ravenscroft (2023) that a more nuanced understanding of CVI that goes beyond the traditional application of the two-stream model could provide better insights, especially in cases where visual recognition challenges occur in the absence of evidence of temporal lobe dysfunction or pathology. Future research focusing on the interconnectivity of these networks, probing in depth the nature of the related visual experiences could well prove valuable in refining both diagnosis and the salient support strategies for individuals with CVI.

The posterior parietal lobes can be considered a central hub for VPDs, particularly in children with CVI. Research, such as that by Williams et al. (2021), suggests that children with VPDs most commonly exhibit atypical posterior parietal processing. Affected children fall into different groups based on the severity and extent of their learning difficulties. The first group, as described by Lueck and Dutton (2015), presents with mild difficulties. The second group shows more severe impairments, likely extending to the middle temporal lobes where motion is processed, leading to conditions such as dyskinetopsia or, in severe cases, akinetopsia. The third group, characterized by research from Little and Dutton (2014), exhibits more profound impairments, including reduced visual acuity and narrowed visual fields. All three groups tend to exhibit lower visual field impairments affecting both eyes, which vary in severity, correlating with the extent of the affected brain regions. This impairment occurs because the visual information from the lower visual field travels through the posterior parietal lobes in the form of optic radiations, where it may be disrupted before reaching the occipital lobes. A comprehensive understanding of the varying combinations of CVIs, including VPDs, allows for better-targeted interventions. For instance, group one may experience simultanagnostic vision and optic ataxia with lower visual field impairments, while group two may also exhibit additional dyskinetopsia. Group three, the most severely affected, may additionally suffer from reduced contrast sensitivity and visual acuities. The range of difficulties observed across these groups suggests a CVI spectrum, with increasing severity of visual and learning difficulties correlating with the extent of brain involvement (Table 1). This spectrum approach also opens the door to new measurement strategies for assessing the effectiveness of support, potentially using percentile-based scales.

**Table 1:** Groups of children with CVI due to posterior parietal lobe involvement.

Groups of children with CVI		
Typical visual acuity		Reduced visual acuity
Working at academic level expected for their age.	Cognitive challenges, behind at school.	Profound visual impairment, often with multiple disabilities

Suggested underlying cerebral visual impairments		
Lower visual field impairment (mild) Simultanagnosia Optic ataxia	Lower visual field impairment (moderate) Simultanagnosia Optic ataxia Dyskinetopsia	Lower visual field impairment (severe) Simultanagnosia Optic ataxia Dyskinetopsia Reduced contrast sensitivity Reduced visual acuity

The research conducted by The Laboratory for Vision Neuroplasticity and others has significantly advanced our understanding of VPDs, particularly concerning motion perception, visual attention, scene complexity, navigation, and recognition difficulties. Children with VPDs often display specific behaviors, such as darting eye movements, slower visual processing in complex scenes, impaired search performance, and difficulty with visually guided movements, which are considered to be due to issues with the dorsal ‘where’ visual pathway (Manley et al., 2023; Bennett et al., 2018; Zihl and Dutton, 2015; Manley et al., 2022; Goodale and Milner, 2013). These challenges, coupled with increased frustration and anxiety (McDowell and Budd, 2018), underscore the need for tailored support based on a clear understanding of the underlying brain processes involved. In summary, while recognition difficulties are common among children with VPDs, they may not necessarily stem from an issue with the ventral ‘what’ visual pathway. Instead, these issues may arise from atypical processing in the posterior parietal lobes. Understanding the specific causes of these difficulties is crucial for providing effective support and ensuring that interventions address the root causes of the problem, not just the symptoms. If not accurately understood, this could lead to the child receiving incorrect or inaccurate support.

While it is important to have this scientific/medical understanding of VPDs, in order to truly help children and adolescents, it also seems imperative to deepen this understanding by hearing from those with the lived experience on how these visual issues actually impact them on a daily basis. Fortunately, with this increased focus on CVI and VPD in recent times, more and more children, adolescents and adults are starting to speak up and share their experiences in blogs, news stories, videos and via social media platforms (McDowell, 2024; CVI Scotland, 2024b; ABC News, 2022; Bartiméus, 2019; Koninklijke Visio, 2018; 1News, 2022). In these accounts, people with CVI share specific issues they have at school, such as ‘*I cannot find my friends*’, ‘*I need more time for most activities*’, ‘*I find it hard to follow the line I am reading*’ and ‘*Playing sport is really hard*’ (Bartiméus, 2019). They also describe the impact CVI has on their day to day life, ‘*When there are a lot of people in the room and it is noisy, I have to leave the room*’, ‘*If I have to spend a busy day searching and looking and everything, it gets a bit worse and I have a harder time looking for things*’ (Bartiméus, 2019), ‘*My vision is actually like a puzzle. In fact, everything is. At school it is quite tiring*’ (Koninklijke Visio, 2018), ‘*I can only see one thing at a time and the more stressed I get, the fewer things I can see*’, ‘*Social interactions can be challenging. When people greet me, I cannot see them clearly*’ (ABC News, 2022) and ‘*I am clumsy and I trip over a lot*’ (1News, 2022). They have also shared emotional accounts of the toll CVI has had on their lives, ‘*I always felt dumb*’ (1News, 2022), ‘*All my life, I have been inventing excuses for not having to participate in things. Because of this, I am often not asked to come along anymore*’ (Koninklijke Visio, 2018). ‘*Other kids always think I am blind, or I am half blind. But I’m not. I am not blind*’ (Bartiméus, 2019), and ‘*It was such a relief to learn that I had CVI. It helped me to realize that it is not my fault. There is a reason why things are like they are*’ (1News, 2022). These are typical accounts of CVI that can inform understanding and help guide best practice approaches for supporting children and adolescents with VDP’s by hearing first-hand how their CVI impacts their daily lives.

### 2.1 Defining visual impairment in children and adolescents with visual perceptual difficulties

A significant challenge in supporting children and adolescents with VPDs associated with CVI is the lack of comprehensive visual impairment classification. Traditionally, the International Classification of Diseases (ICD) has primarily focused on visual acuity for classifying vision impairment (World Health Organisation, 2019). This approach often fails to identify individuals with typical or near typical visual acuity but significant VPDs. However, the ICD-11 has made strides in recognizing a broader spectrum of visual impairments. Specifically, it includes the following relevant codes:

- 9C43—Visual impairment with higher visual processing dysfunction
- 9C44—Visual impairment with combined structural and higher visual processing dysfunction
- 9C4Y—Other specified visual impairment
- 9C4Z—Visual impairment, unspecified

These codes represent a significant step forward in acknowledging VPDs and other complex visual impairments that may not be captured by traditional acuity measures. However, for the future, it is clear that specific codes, rather than those labelled “unspecified,” will become necessary to secure remuneration for medical and habilitation service providers.

Despite this progress, challenges also persist in the practical application of these classifications, particularly in educational settings. For children and adolescents with VPDs, the lack of a clear, widely recognized diagnosis can still impact their access to educational support and funding. Many education systems continue to rely on more traditional definitions of visual impairment, which may not fully capture the needs of students with VPDs. This gap in recognition and support puts affected children at a disadvantage compared to peers with more easily identifiable visual conditions (such as albinism or nystagmus). The discrepancy can lead to inadequate educational interventions and reduced access to necessary habilitation or rehabilitation services. Moving forward, it is crucial to:

- Increase awareness of the ICD-11codes related to VPDs among medical professionals and educators.
- Develop standardized assessment tools that can accurately identify and classify VPDs.
- Update educational policies to recognize and accommodate students with VPDs, even when traditional visual acuity measures are within typical ranges.
- Agree on a unifying definition of the different types of CVIs so that they can map directly on to the ICD classification system.
- Promote research into effective interventions and support strategies for individuals with VPDs.

By addressing these areas, we can work towards a more inclusive and supportive environment for children and adolescents with VPDs, ensuring they receive the appropriate diagnosis, educational support, and interventions they need to thrive.

### 2.2 Current challenges in the assessment of visual perceptual difficulties

Another problematic area in the management of children and adolescents with VPD is around the assessment and diagnosis process for CVI. Due to the heterogeneous nature of CVI, the diagnosis is often missed in many children, especially in those with typical or near typical visual acuity (Lowery et al., 2006; Chandna et al., 2021). This was highlighted by McDowell (2020b) in their research looking at the impact that knowledge had on empowering parents of children with CVI. Reports by the 75 parent participants in the research showed it took an average of 3.6 years for a CVI assessment and diagnosis, with the longest length of time being 18 years. Many more stories of lengthy delays in diagnosis, or ongoing fights to get a diagnosis have been shared anecdotally with the authors personally and with others via social media platforms. Ideally, the optimum time for CVI to be identified and diagnosed is in the early years (under 5). However, while some early years intervention programs (for children aged under 5 years old) are aware of CVI many are not. More often than not, a child with CVI and VPDs does not get identified until they have started school, when the difficulties with learning become more apparent in formal learning settings. Another roadblock for many in terms of getting a CVI diagnosis, is the reliance on evidence of injury or damage to the visual brain on neuroimaging, specifically an MRI. However, in one study of 33 children diagnosed with CVI (who all had typical or near typical vision), 23% showed none of the reported MRI findings related to this condition (Chandna et al., 2021), suggesting that up to one quarter of children with CVI may not have evidence of brain injury on neuroimaging and further assessment needs to be undertaken to confirm the diagnosis. Further research needs to be conducted on a larger group of children from all three of the groups outlined by Lueck and Dutton (2015) to confirm these findings.

Even if a child has been identified as potentially having CVI, there is still ongoing debate and little consensus on what further assessments should be conducted. In addition, generally the focus of assessment is to diagnose CVI, which does not allow for evaluation of improvements in visual functioning following interventions or help to identify what specific education support is needed. Assessments covering both purposes would significantly help the management of children with CVI. A number of recent publications have attempted to provide a best practice approach for the assessment and diagnosis of CVI, proposing a multidisciplinary approach that includes medical history and CVI question inventories and questionnaires, ophthalmological and orthoptic assessment, assessment of visual behaviors and direct observation, visual perception tests, ocular movement and posture assessments, neuropsychological assessment, neurodevelopmental tests, neuroradiological evaluation and magnetic resonance imaging, genetic assessment, IQ assessments and clinical electrophysiology (McConnell et al., 2021; Boonstra et al., 2022). However, in many countries, this full battery of assessments would be cost inhibitive and could only be conducted on children or adolescents where it had been predetermined that a CVI diagnosis was very likely. To help with this, Pilling et al. (2022) have proposed a checklist approach for determining whether a child needs a full CVI assessment or not, which can be used by general practitioners or first line optometrists. Within the checklist approach, CVI may be present if two or three of the following criteria are met: (1) presence of risk factor(s), (2) reported or observed atypical visual behaviors, and (3) verifiable visual dysfunction on examination. The authors also outline that CVI is highly likely to be present if all three criteria are met (Pilling et al., 2022). To further support this checklist approach, the authors provide information on what would be classed as high-risk factors, including children who:

- are born premature,
- and/or hypoxic ischemic encephalopathy,
- and/or have cerebral palsy,
- and/or developmental delay,
- and/or down syndrome,
- and/or hydrocephalus.

The authors also outline that atypical visual behaviors may include (but are not limited to) non refractive error reduced binocular visual acuity (presenting as lower visual function impairment), abnormal fixation, visual field deficit or inattention, oculomotor impairments (jerky or smooth pursuits or inaccurate saccades) and VPDs. This checklist approach again highlights the need for effective, accessible validated VPD assessment tools to enable clinicians to confirm the suspected CVI diagnosis following examination.

However, while the majority of children affected by VPDs may present with reported or observed atypical visual behaviors, distinguishing between difficulties caused by CVI and those arising from other conditions such as autism, ADHD, dyslexia, or developmental coordination disorder remains challenging (Chokron et al., 2021). This difficulty is particularly pronounced in young preschool children and those with comorbid conditions, where overlapping symptoms can obscure accurate diagnosis. Therefore, effective methods to identify children with visual issues that specifically differentiate CVI from other conditions are essential. For this, structured clinical history taking emerges as the most effective assessment tool in this context. Unlike standardized questionnaires, it allows for a comprehensive and individualized understanding of the child’s visual experiences and behaviors. Chandna et al. (2021) conducted an in-depth evaluation demonstrating the high efficacy of this approach in identifying potential CVI. Their findings highlight that a detailed clinical history can capture subtle visual processing difficulties that may not be evident through observational methods alone, making it unparalleled in its ability to discern CVI-related issues.

While tools like the 5 or 11-question inventory have been used as effective screening processes for identifying children with potential CVI, they have limitations in specificity and may not fully capture the complexity of each child’s condition (Gorrie et al., 2019; Chandna et al., 2021). These inventories include specific questions to help identify children with possible lower visual field impairment, a condition that is relatively straightforward to detect and difficult to attribute to other causes. The presence of a lower visual field impairment can indicate additional VPDs, but relying solely on questionnaire responses may not provide the depth of information needed for accurate diagnosis, especially in children with comorbid conditions. Additionally, the L94 Visual Perceptual Battery developed by Ortibus et al. (2009) offers a structured method to assess visual perception in children with CVI. Further studies on the L94 Visual Perceptual Battery have validated its utility, showing that it can effectively identify visual perception deficits in children with complex developmental profiles (Ortibus et al., 2011).

An ongoing challenge is the effective assessment of children who have a combination of difficulties or who are non-verbal. Traditional assessment tools often rely on the child’s ability to follow instructions and answer questions, which may not be feasible in this population. This underscores the importance of flexible and adaptable assessment methods. Structured clinical history taking, involving detailed interviews with caregivers and observations of the child’s behavior in naturalistic settings, can provide valuable insights when direct communication is limited. In conclusion, while various assessment tools are available, structured clinical history taking remains the most effective method for identifying CVI in children, particularly those with comorbid conditions (Chandna et al., 2024; Dutton and Jacobson, 2001; Philip and Dutton, 2014). It allows clinicians to gather comprehensive information tailored to each child’s unique presentation, thereby enhancing diagnostic accuracy and informing appropriate interventions. Incorporating tools like the L94 Visual Perceptual Battery can further support the assessment process by providing evidence-based methods to discern visual processing difficulties, although their limitations should be acknowledged.

Another common method for identifying children with visual issues is through national screening programs, which are often undertaken prior to children starting school at age 5 (Muller et al., 2019). However, as the focus of this screening is mainly on visual acuity, children with VPDs are not identified as having perceptual visual issues and therefore, no further assessment is conducted. This was highlighted by McDowell and Butler (2023) in their research to validate the Austin Assessment (a screening tool for CVI related visual issues). The Austin Assessment was used to screen 271 children in one New Zealand primary school and following further assessment by an ophthalmologist on the children who were flagged in the assessment, 17 were found to have ‘a verifiable reason or clinical finding for a positive Austin Assessment result’. In reviewing the children’s records and talking with their parents, none had been picked up in the B4 screening process as having a visual issue (New Zealand’s vision screening program). This could be easily remedied by simply including CVI screening tools, for example the 11 Question Inventory and the Austin Assessment, in vision screening programs (Chandna et al., 2021; McDowell and Butler, 2023). While further research is needed, initial reports show that together, the 11 Question Inventory and Austin Assessment provide objective and subjective evidence of VPDs that can then be confirmed (or not) following a subsequent CVI specific assessment process (McDowell and Butler, 2023).

### 2.3 Impact of visual perceptual difficulties on learning and development

The impact VPDs can have on a child’s learning and development can be significant, especially if they do not receive early intervention services, as these can impact how a child accesses and processes learning material and experiences. Some of the common challenges experienced by children with VPDs include struggling to stay focused on learning tasks, being easily distracted by competing sensory information in the classroom environment, finding it difficult to copy or to keep up with information being presented in the classroom, being overly sensitive and startling often to movement and noise in the classroom, accompanied by high levels of visual and overall fatigue (Lam et al., 2010; Philip and Dutton, 2014; McDowell, 2021). For many children with CVI, this can then lead to learning difficulties in specific subjects that may require additional learning support in the classroom. While there has been limited research in this area, two studies have shown that the number of children experiencing learning difficulties could be as high as 80% (Williams et al., 2011; McDowell, 2023). Both papers reported that the common learning difficulties experienced by children with CVI included underachievement in reading, math and writing (Williams et al., 2011; McDowell, 2023). These issues are often compounded by disparities in the child’s functioning (both visually and cognitively) over the course of the school day. Many external (clutter, noise, movement) and internal (fatigue, hunger, sickness, anxiety) factors can impact on a child’s visual abilities, which results in children performing less well in vision related tasks in some situations, as compared with others. This disparity is often misunderstood by the adults around the child, who may incorrectly conclude the that child is uninterested in learning, is lazy or is choosing not to engage in the learning process (Erasmus, 2015). In addition to learning difficulties, children with CVI often experience difficulties with developing and maintaining friendships, high levels of anxiety and an inability to regulate emotions effectively, as well as issues with being able to move around their environment safely and confidently (McDowell and Dutton, 2019; McDowell, 2019; McDowell, 2023).

Together, these issues can greatly impact on a child’s physical and mental wellbeing, especially when they see themselves as being different from their peers. In particular, the challenges in developing and maintaining friendships causes significant distress for many affected children. This was highlighted in the research by McDowell (2023) focused on understanding and supporting children with CVI in mainstream classrooms. Both parents and teachers involved in the research reported children sharing with them how lonely they felt and how hard it was for them to not have any friends. Similar experiences have also been shared by both children and adults with CVI, through blogs and social media platforms. The high levels of anxiety and difficulties with emotional regulation can also impact significantly on the lives of both the child and the people around them, especially if the root causes of the anxiety are not well understood. Both parents and teachers have reported children seeming to switch from being relaxed and happy 1 min, to being incredibly distressed and inconsolable in an instant with no apparent reason for the sudden change (McDowell, 2020a; McDowell, 2023). While not reported in the literature, many with the lived experience (children, parents, adults) of CVI have also reported physical health issues such as headaches and digestive issues that they can directly relate to the visual issues (CVI Society, 2024; McDowell, 2024). This is another area that requires further examination and research to ensure appropriate supports can be put into place to reduce these issues.

### 2.4 Awareness of CVI and visual perceptual difficulties in education settings

In recent times the numbers of children in mainstream education with learning support needs has increased significantly. While it is difficult to determine exact figures (due to differences in identification and reporting between different conditions and countries), in New Zealand, it has been estimated that 15–16% of students have additional learning needs (Wylie and MacDonald, 2020; Bourke et al., 2021). This is compared with between 20 and 30% in England (Bourke et al., 2021) and 37% in Scotland, where numbers have increased from 37,000 in 2007 to 260,000 in 2023, representing over a third of all children in schools in Scotland (Scottish Government, 2023). The reason for this increase is in part due to changes in education policies and better identification and assessment practices for developmental conditions such as autism, attention deficit hyperactivity disorder (ADHD), fetal alcohol spectrum disorder (FASD) and dyslexia (Bourke et al., 2021; Yu et al., 2023; Ministry of Education, 2007; Ministry of Education, 2019). This rise in students with additional needs in mainstream education settings has meant that teachers have had to expand their knowledge base around these conditions, which is often done by engaging in professional learning opportunities (Bonne and Wylie, 2017). While further work still needs to be done in this area, the majority of classroom teachers at least feel confident in working with students who have additional needs who require learning support (Bonne and Wylie, 2017; Wylie and MacDonald, 2020; Nicholson and Dymock, 2015), especially following professional development in specific areas, such as autism (Kossewska et al., 2022), ADHD (Zentall and Javorsky, 2017) and dyslexia (Bell, 2013).

Unfortunately, CVI has not been part of this trend, with children and adolescents with CVI and VPDs likely not included in the prevalence data of students with learning support needs for each country, due to the fact that many are undiagnosed and/or currently do not meet the requisite standards for an atypical visual impairment classification. In addition, CVI is still a largely unknown condition in mainstream educational settings. Recent research by Jayasinghe (2023) found that 98% of primary teachers and 80% of secondary teachers across the UK had never heard of CVI. This is similar to the findings of McDowell (2023) in their research on supporting children with CVI in mainstream classrooms in New Zealand, where all 11 of the classroom teachers involved in the research had never heard of CVI. This lack of knowledge on one condition, yet with a more expanded knowledge base on other developmental conditions is further exacerbating the issue of children and adolescents with VPDs not receiving the support they need. Due to the behaviors children with CVI often display in classrooms settings as a result of their VPDs, for example, difficulties with math and reading, struggling to focus, being easily distracted, difficulties with emotional regulation and social interactions (Lam et al., 2010; Philip and Dutton, 2014; Williams et al., 2011; McDowell, 2023), they are often mistakenly identified as having other conditions such as autism, ADHD, dyslexia and auditory processing disorder (Pawletko et al., 2015; Dutton, 2015).

This has been highlighted in the research conducted by McDowell and Butler (2023) to validate the Austin Assessment. When the list of children identified as potentially having visual issues (due to a positive Austin Assessment result) was presented to the school involved in the research, the school Special Education Needs Coordinator (SENCO) and classroom teachers all reported that they ‘knew something was going on with each child’. However, they had not even considered it was the child’s vision that was causing the learning difficulties they were seeing in the classroom. Instead, owing to their expanded knowledge base around other developmental conditions, they had attributed the issues to either autism, ADHD, dyslexia or auditory processing disorder. While many children may have these conditions in addition to CVI, as highlighted by Gorrie et al. (2019) in her research looking at the effectiveness of CVI question inventories, which found that virtually all children who potentially had CVI also had been labelled as having other conditions including autism, ADHD, dyscalculia, dyslexia, dyspraxia, ocular visual impairments, deafness, hearing impairment, and intellectual disability. Children with multiple conditions still need appropriate support for their CVI in addition to appropriate interventions of the other conditions.

In contrast, research by Pilling et al. (2022) found that specialist vision teachers, referred to as Teachers of Pupils with Vision Impairment (TVI) are well aware of CVI and do have some understanding of the visual issues associated with CVI and how to support a child in different education settings. This is likely due to an increase focus on CVI in professional learning spaces in recent years and the creation of web-based resources such as the CVI Scotland website (Ravenscroft et al., 2021). Unfortunately, it seems that this increased knowledge and understanding of CVI amongst TVIs is not being disseminated to mainstream teachers, suggesting that it is not just enough to focus on upskilling TVI’s, there also needs to be a targeted approach to teaching mainstream teachers. For children in groups two (who face learning difficulties) and three (who are profoundly affected) of the CVI categories outlined by Lueck and Dutton (2015), due to their additional education support needs, they may already be in a special education placement or receiving additional learning support within a mainstream setting. Depending on the school setting (i.e., a specialized school of the blind and visually impaired compared with a more generalized special school), teachers and support staff may or may not have any awareness and understanding of CVI. This highlights the need for a specific focus on CVI to be a mandatory component of tertiary training programs for Vision and Specialist teachers who work in vision and special school settings.

### 2.5 Current approaches to supporting children and adolescents with visual perceptual difficulties in the classroom

Until recently, owing to a paucity of research focused specifically on supporting children and adolescents with VPDs in the classroom, many were being supported using approaches that had been shown to be effective for children with ocular visual issues (OVI) (Martin et al., 2016; McDowell, 2021). Examples of this include, enlarging printed material, using magnification devices and having children sit in locations in the classroom that faces them towards cluttered and busy spaces (to bring them closer to the information). While this may help some children with CVI, for others it may make it even more challenging to access the learning material (Martin et al., 2016; McDowell, 2021). Due to the difficulties with visual crowding and clutter, it is important to reduce the amount of visual information a child with visual perceptual difficulties needs to process at any one time. Enlarging the material, or bringing a child closer to the front of the classroom, simply increases the visual information and makes the visual scene even more complex (McDowell, 2024). With this understanding, vision educators are starting to move away from using OVI strategies in the classroom, instead exploring other options that could be more effective for children with VPDs. For example, decluttering learning spaces and material, introducing calm breaks and teaching emotional regulation strategies (McDowell, 2020a; McDowell, 2023; Hokken et al., 2024). However, it is important that this trend is supported by the use of validated, effective approaches that take into account the unique visual considerations of children and adolescents with VPD. Currently, as we have shown, all three of our groups are not getting the support they need in school, to the potential detriment of each affected child.

## 3 Discussion

While there have been some improvements in recent years, overall, the current approach to supporting children and adolescents with VPDs in schools is not, as a whole, working. This is highlighted by the challenges many children with these visual issues in mainstream classrooms are still experiencing, including learning difficulties, struggles with friendships and social interactions, challenges with emotional regulation and mobility issues (Williams et al., 2021; McDowell, 2023; McDowell, 2019; McDowell and Dutton, 2019). In others, learning is so severely affected that they need special educational placements. While these issues in themselves make navigating the school day incredibly hard for children with CVI, without the right support, this could also lead to additional mental health issues that impact greatly on overall health and wellbeing (Goodenough et al., 2021; University of Bristol Research, 2019; McDowell, 2019). Historically, decisions around the management of children with CVI, from identification to assessment, diagnosis and support have been based on external factors, such as funding (both in health and education), limitations in current systems (in both health and education) and current understandings of CVI and VPD (from the research). This approach needs to be re-evaluated with a more collaborative agenda where different voices in the community, including researchers, practitioners and professionals, parents and children with the lived experience considered. In this way, decisions are based on evidence-based practice, where the intersection between the three circles of evidence (research evidence, practice evidence and lived experience evidence) guides a best practice approach (Bourke et al., 2005). An evidence-based practice approach will also provide an opportunity to bring the (at times) competing views of the health and education systems together to ensure every child and adolescent with VPD around the world has equal opportunity to thrive in their education journey.

### 3.1 Identification of visual perceptual difficulties

The first step in the management of children and adolescents with VPD is identification. Currently, the main avenue for identifying common visual issues is vision screening, which is commonly done on all newborn babies, with more comprehensive screening on babies at high risk for visual issues (such as those born premature, infants with cerebral palsy, HIE and other neurological challenges). Further screening is done in most countries at around age 4 and in some countries again at age 11 (i.e., New Zealand). Other avenues include identification by medical practitioners and other professionals such as teachers and therapists and identification by parents and family members. While all of these avenues provide ample opportunities for a child’s VPDs to be identified early, due to the lack of awareness of CVI across the general public, they are not being utilized effectively. To help with this, frontline medical providers, including general practitioners and optometrists need to have a good understanding of CVI, including the range of visual issues associated with this condition, how they might present, and the common manifestations and behaviors demonstrated by children at different developmental stages. For example, in young children this might present as showing signs of distress in busy, cluttered and noisy environments and not making eye contact with familiar and unfamiliar people. For school aged children, this might present as difficulties with learning to read and write, not learning the same as their peers, difficulties with friendships, avoidance of sporting and physical activity and high levels of anxiety (Williams et al., 2021; Bartiméus, 2019; McDowell, 2023).

In line with this, front line medical providers (GP’s and optometrists), should also have access to validated CVI screening tools, so that when visual issues are observed or parents share their concerns about their child’s vision, functioning or behavior there is a clear first step in the pathway to identification. For this to be effective however, the screening process needs to include a subjective observation of the child’s visual abilities and an objective observation of the child’s visual functioning, for instance combining a question inventory with a screening app or tool. To ensure this approach is manageable and easy to integrate into short consultation timeframes, the screening would need to be able to be done in under 5 min and clear next step guidelines outlined for medical providers and parents (Legge, 2024). This combined approach to CVI screening could also be included in vision screening programs for school aged children to expand them from solely focusing on visual acuity, to also including visual perceptual difficulties. This approach has been shown to be effective in one primary school in New Zealand as part of the research to validate the Austin Assessment (McDowell and Butler, 2023). Again, clear next steps would need to be in place to ensure that any child flagged in the vision screening process had a clear pathway for further assessment and possible diagnosis of CVI.

Another avenue for identification, especially in the children whose VPDs are impacting them in an education context but may not be severe enough for a CVI diagnosis (under the current thresholds), is the school itself. Teachers and other learning support staff, such as Special Education Needs Coordinator’s (SENCOs) are often the first to observe learning difficulties or different behaviors in children or are the first people parents raise their concerns about their child with. Simple to use, effective screening tools that help to identify potential VPDs could be used in these situations, not for the purpose of obtaining a diagnosis, but instead, for the purpose of guiding education support. For instance, the initial 11 Question Inventory followed by the 39 Question Inventory could be used to determine the main challenges for the child (Dutton et al., 2010; Houliston et al., 1999; Chandna et al., 2021; Hellgren et al., 2020).

If a diagnosis was needed for education funding and support services, the same pathway of being seen by a frontline medical provider (GP, optometrist, pediatric ophthalmologist) could be used. However, for schools to be able to help with identifying children with VPD, they firstly need to be aware that these visual issues exist in the first place. With the increase in children with additional learning needs in recent times, teachers have already had to expand their professional knowledge base to include identifying and supporting children with a range of developmental conditions, including autism, ADHD, dyslexia and auditory processing disorder (Bonne and Wylie, 2017; Wylie and MacDonald, 2020). CVI and VPD could easily be added to this by providing professional development opportunities to teachers and learning support staff on the specific visual issues, how they often present and the impact they have in a learning environment. This approach has been shown to be effective for other conditions, including autism, ADHD and dyslexia (Kossewska et al., 2022; Zentall and Javorsky, 2017; Bell, 2013) and could be just as effective for CVI.

Another group that could help with the identification of VPDs are parents. As the experts on their child, they see the daily impact of the visual issues both at home and at school. This was highlighted in the research by McDowell (2023) on supporting children with CVI in mainstream classrooms. Parents involved in this research shared that they had known something was going on with the child’s learning but could not quite put their finger on what the issue was. Many had explored other conditions such as dyslexia, but were unsure about how to go about identifying what was going on (McDowell, 2023). This was similar to the finding by Gorrie et al. (2019) in their research that assessed the effectiveness of two CVI question inventories on children who did and did not have CVI. CVI was indicated in many who had not been identified as having CVI (55 of 431 using one question inventory, 166 of 431 using the other question inventory). However, the vast majority had already been identified as having other conditions, including dyslexia, autism and ADHD. Although the research did not explore how the children had been identified with these conditions, it could be surmised that the increased knowledge about developmental conditions in recent years, led to parents recognizing possible characteristics of these conditions in their children and seeking out further information and support, which in turn led to a diagnosis.

Empowering parents, by providing access to information about CVI and a simple, easy to use, effective CVI screening tool has the potential to significantly improve the identification of children who have these visual issues, which would in turn ensure they receive the support they need. This approach has been shown to be effective for one family in New Zealand, who used the Austin Assessment with their 13-year-old son who had been diagnosed as having learning difficulties. They had been recommended to do further vision assessment; however this was not easily accessible to the family and cost prohibitive (Austin Assessment, 2024). Watching their child do the Austin Assessment and the results shared in the report, helped them to see exactly what was going on with his vision and how to help him at home and at school. Empowering parents in this way, will also ensure parents feel confident in supporting their child and are able to take a central role in any education support plan (McDowell, 2020b).

### 3.2 Assessment of education needs for children and adolescents with visual perceptual difficulties

From research, it is evident that the number of children with CVI and VPDs is significant, with some estimates indicating it may affect approximately 3.4% of children (Williams et al., 2021). However, it is important to note that this figure is based on a single study, and as yet, there has been no replication. Caution should be taken in how widely we generalize these findings until further research is conducted. Moreover, the number of children affected by CVI is expected to rise, partly due to medical advancements in treating conditions associated with CVI (Good et al., 1994; Chong and Dai, 2014). This issue is not restricted to high-income countries; the increase is also being noted in low-and middle-income countries (LMICs). Factors such as poor maternal care leading to hypoxic–ischemic encephalopathy (HIE) and the increasing number of neonatal intensive care units (NICUs) in urban centers, resulting in more premature neonates with periventricular leukomalacia (PVL), contribute to this rise. However, healthcare systems and education infrastructures in these regions are often overstretched, underfunded, and not fully equipped to manage these challenges. Additionally, babies in LMICs are more likely to be exposed to *in utero* infections and pre-natal brain injury, which can further contribute to the prevalence of CVI (Ssentongo et al., 2021; Paneth, 2021; Houweling et al., 2007; Patial and Swaminathan, 2018).

The growing number of children with CVI has also raised concerns about overburdening already strained public health systems in developed countries (McDowell and Butler, 2023). However, if decisions and policies focus solely on economic factors, there is a risk that some children and adolescents with VPDs could be overlooked, especially when traditional definitions and diagnostic criteria are rigidly applied. It is important to recognize that these more “traditional” definitions, while potentially viewed as strict, are often shaped by diagnostic conventions that leave little room for flexibility. As a result, children who are functioning at a high level, despite their visual impairments, may be missed as they are seen to be coping within the expected norms, although they may not have reached their full potential. One alternative is to focus on educational needs rather than solely on visual deficits when assessing children. By integrating this approach into early intervention programs, it may be possible to ensure children receive the necessary support early in their development, reducing the demand on public education services in the long term. This could also lower the lifetime economic costs associated with ongoing support. However, this approach carries a downside: children who work hard to overcome their visual impairments may be overlooked, as their high performance may mask their struggles. Further consideration is needed on how best to support children with CVI to reduce the impact of this constant ‘over functioning.’

To support the assessment of educational needs, an evidence-based approach can be adopted, drawing on the expertise of researchers, practitioners, and families (Bourke et al., 2005). Once a child is identified as potentially having CVI or VPDs, a comprehensive assessment should be conducted to develop an individualized CVI profile. However, this need not require lengthy medical appointments; comprehensive history-taking inventories have proven effective in identifying specific CVIs in children (Hellgren et al., 2020). These inventories rely on parents and, in some cases, the child, to report daily challenges. Since parents observe their children most closely, they can provide valuable insights into their child’s functioning (Pueyo et al., 2014). Parents can complete the inventories at their convenience, allowing specialists to review the information before appointments, which can streamline the assessment process. This approach empowers parents to take an active role in their child’s education and support plan (McDowell, 2020b). However, while empowering parents can be positive, there is also a risk that providing limited information might lead to overconfidence or unrealistic expectations. A balance is needed to ensure that parents are well-informed but also aware of the complexities of their child’s condition.

In addition to history-taking inventories, percentiles can be introduced into CVI assessments to objectively determine where a child sits on the VPD spectrum compared to peers (Hellgren et al., 2020). However, subjective assessments are equally important (Chandna et al., 2021). Currently, there is no easily accessible standardized tool for accurately identifying and classifying VPDs, and developing such tools should be a priority for the research community. Clinicians require access to assessment tools that extend beyond visual acuity tests to ensure they can effectively evaluate children with VPDs. Moreover, the role of vision education and therapy practitioners must be considered. Teachers of the visually impaired (TVIs) play a key role in conducting functional vision assessments (FVAs), which assess a child’s visual abilities in real-world settings, such as classrooms, rather than clinical environments. Incorporating validated VPD-specific tools into FVAs would help ensure that children receive appropriate interventions. Regular assessments are needed to determine the best way to present learning materials and optimize learning environments for each child. To implement effective FVAs and clinical assessments, clinicians and practitioners must have a thorough understanding of VPDs, highlighting the importance of including CVI in tertiary training programs.

Classroom teachers also play an important role in assessing a child’s educational needs. History-taking inventories can be useful here as well, as teachers may observe different challenges in school compared to those seen at home, where the sensory environment is less complex. This was evident in McDowell (2020a) case study, where a parent observed, “*At home she is like a typical child. She can do almost everything her siblings can do. But when she gets to school, she becomes this hugely disabled child who appears so different to her peers. I just wish the school got to see the child we see at home*.” Teachers often notice fluctuations in a child’s visual functioning and behavior over the course of the school day (McDowell, 2023) and can provide valuable insights into the accommodations and strategies that might be needed at different times and in varying contexts. A collaborative, multidisciplinary approach that integrates clinical, practitioner, educator, and parental input can create an evidence-based assessment of a child’s educational needs. This approach ensures that the child receives the most appropriate support while reducing the burden of lengthy and costly assessments on any one service. By working together, all stakeholders can help minimize learning and developmental challenges, including social, emotional, and physical health issues, ensuring that each child has the best opportunity to succeed in their educational journey.

### 3.3 Education support

For any education support to be effective for a child or adolescent with CVI and VPDs, it needs to be understood that they will have their own unique presentation of visual issues (even in children with the same underlying cause) (Hyvärinen, 2019). In addition, the impact these visual issues have on them in a classroom environment will depend on personality, life experiences and natural abilities. Traditionally each child would go through a thorough assessment to determine their individual education needs. However, there are a few overarching principles that should be considered when developing individual education plans for any child or adolescent who has VPDs. The first of these is to reduce visual clutter in the learning environment, including the learning material (i.e., worksheets, online material and in books) (Hokken et al., 2024). The importance of this was highlighted by an adolescent with CVI in a blog on effective support, where they explained ‘*What I tell some of my teachers is to make it as boring as they think they possibly can, and then make it even more boring*’, as this approach results in the material being accessible for them (CVI Scotland, 2024b). For many children with CVI, the main barrier in accessing and processing learning material is the amount of visual clutter in their classroom environments. Research by Little and Dutton (2014) and McDowell and Budd (2018) has shown that when this clutter is reduced or blocked out for children with severe CVI (including reduced visual acuity) in special school classrooms, it significantly improved the child’s learning, behavior and visual functioning. This approach of reducing visual clutter within the learning environment was also shown to be effective for children with VPD in mainstream classrooms in recent research by McDowell (2023). In this research, classroom spaces, such as surrounding the large TV screen (where all learning material was displayed), the teacher’s desk and classroom walls were decluttered for a period of 10 weeks. The teachers involved in the research reported that they noticed improvements in engagement and levels of achievement in the children who had been identified as having VPDs. When the children were asked if they liked the decluttered spaces, their comments included statements such as ‘*There is less noise in my brain when the classroom is not so cluttered, and it is easier to think*’, and ‘*My head does not hurt as much at the end of the school day when there is not so much stuff in the classroom.*’

Another important overarching principle for supporting children and adolescents with VPD in the classroom is ensuring everyone involved in supporting the child, including the child themselves, understands the visual issues and the impact they have in an education setting. This can be achieved by providing individualized CVI education sessions for parents, teachers and other professionals working with a child with visual perceptual difficulties. This approach has been shown to be effective by McDowell (2020a) and McDowell (2023) in research with children in mainstream education settings. The education sessions were provided after a thorough assessment of the child’s education needs had been conducted and included an outline of the specific visual issues the child had, how they impacted them in the classroom and the individualized approaches that were needed to support the child. CVI simulation (CVI-SIM) videos (CVI Scotland, 2024a) were also used to help show what the world might look like for the child. Both parents and teachers have reported that these sessions really helped them to understand what the world looked like for the child (McDowell, 2020a). One teacher even noted *‘as soon as I could empathize with what life was like for them in the classroom, suddenly it became very clear how to help*’ (McDowell, 2023). The CVI education sessions and sharing of the CVI-SIM videos can also help the child themselves, to understand their own visual issues better. Children have shared with the authors anecdotally that they can identify with the CVI-SIM videos, as they show what they experience in everyday life. Sharing these videos with their teachers helps to ensure the teacher then understands why certain learning activities can be so challenging for them.

Another consideration many with the lived experience of CVI and visual perceptual difficulties are requesting (both adolescents and adults), is the opportunity, where appropriate, to learn blindness strategies from a young age such as braille and Orientation and Mobility techniques (i.e., the use of a mobility cane or guide dog). Currently, most educational approaches introduced for children with CVI require the use of the child’s vision (CVI Scotland, 2024b). However, for many, the constant strain and over functioning required to be able to access the learning material equal to their peers with typical vision over the course of the school day causes significant visual and overall fatigue that often reduces their access to their vision (Bartiméus, 2019). This was shared so eloquently at a recent conference by four adults sharing their perspectives in a lived experience panel. All four described situations where the visual scene was so overwhelming, that it was easier to switch off their vision and use their other senses to access information and navigate their surroundings (Andrésdóttir et al., 2024). As one of the panelists described:

‘*I have a limited amount of energy supplies for the entire day. Certain activities take up significant amounts of this supply, leaving me with even less supplies to get me through other activities over the course of the day. Once I have depleted my daily supply, my visual function significantly decreases, and I have to try to get through the rest of the day with even less vision than normal*’.

A simple solution to overcoming this issue and preserving energy levels for activities where vision is the best option, is to teach alternative non visual options to children and adolescents with VPDs as another tool to have in their toolbox. As noted by Yellowstone in their blog series on the CVI Scotland website ‘*blocking out my vision actually enables me to navigate the school more easily*’ (CVI Scotland, 2024b). However, this approach might not work for all children and adolescents with visual perceptual difficulties, highlighting that the main premise for any approach introduced to help in a classroom and school environment needs to be based on individual needs, appropriately matched to each individual child’s ability level, needs and goals.

## 4 Conclusion

While there has been significant progress in expanding the knowledge base around children and adolescents with VPDs, especially in the last 10 years (based on the number of recent publications in this area), there is still a lot of work to do. For effective and lasting change that will ultimately improve the lives of affected children and adolescents, we believe the community, including researchers, clinicians, practitioners and those with the lived experience (individuals with CVI and VPD and their parents) need to work together to make informed decisions around identification, assessment and education support. For these to be effective, we also need policy makers, including the World Health Organization to better understand CVI and VPD and be held more accountable for improving outcomes for children with CVI. While we offered some solutions and approaches for identification, assessment and education support, we have also identified some key areas where further research is urgently needed. These include:

Creating effective, accessible validated VPD assessment tools (for clinical use and in FVA), that not only help to determine initial needs, but also provide a method for assessing the effectiveness of interventions over time. The use of percentiles, as highlighted by Hellgren et al. (2020) could be further explored for this purpose. It is also important that these tools cater for the wide spectrum of children affected, including those with communication limitations.

Further research on the impact of VPD’s when not supported effectively in an education context and the correlation with the increase in numbers of children with learning support needs that require additional classroom support.

More research on the impact of VPD’s and over functioning (working so hard each day just to access the visual world the same as their peers) on emotional and physical wellbeing and health.

Research on the benefits of the use of non-visual strategies (including braille and O&M strategies) for children and adolescents with CVI.

## 5 Limitations

The aim of this review is to show that despite progress in recent years in the knowledge base around children and adolescents with VPDs, more work still needs to be done to recognize individual needs and to address them effectively. We believe we have provided a comprehensive overview of this topic, bringing together different schools of thought, outlining the main issues and potential solutions. In the conclusion, we also highlight the current research gaps and what we feel needs to be done to address these. What became apparent as we were writing this paper, is the paucity of research in this area, especially in terms of supporting children with CVI and VPDs in school, in a range of different education settings, hence the reliance on only a few papers. To help overcome this, we have included firsthand accounts from people with CVI and those supporting children and adolescents with CVI (teachers, parents), as we believe it is important that these voices are heard and included in any future work to progress the field.
